# Supplementary material for: Pharmacokinetic/Pharmacodynamic Target Attainment Based on Measured versus Predicted Unbound Ceftriaxone Concentrations in Critically Ill Patients with Pneumonia: An Observational Cohort Study
Source: Antibiotics (Basel). 2021 May 11;10(5):557. doi: 10.3390/antibiotics10050557 (PMC8151456; doi:10.3390/antibiotics10050557)
Supplement: Supplementary file 1 [file antibiotics-10-00557-s001.zip › antibiotics-1211732-supplementary/Ceftriaxone_Supplementary materials_Figure S1.pdf]

Pharmacokinetic/Pharmacodynamic Target Attainment Based on Measured Versus Predicted Unbound Ceftriaxone Concentrations in Critically Ill Patients with Pneumonia: An Observational Cohort Study

Supplementary Materials

Figure S1

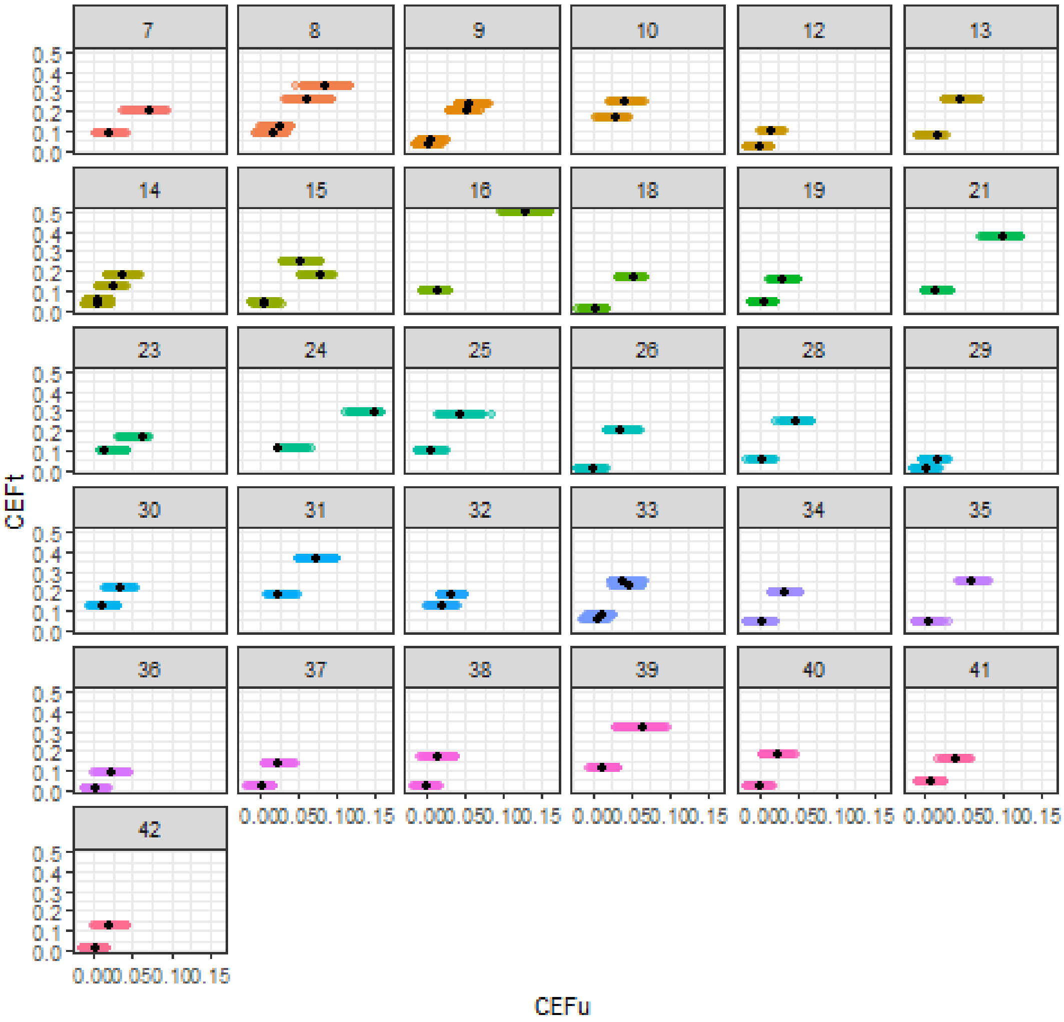

Figure S1. Observed ceftriaxone concentrations (CEFt = total; CEFu = unbound; mmol/L) and visual predictive check of the final protein binding model (Eq. 4) of the 31 patients. The black dots are the observed concentrations. The shaded areas represent the simulated CEFu (n = 1000). The observed concentrations fall within the range of simulated concentrations, hence demonstrating adequate fit of the model.
