# Supplementary material for: Pharmacokinetic/Pharmacodynamic Target Attainment Based on Measured versus Predicted Unbound Ceftriaxone Concentrations in Critically Ill Patients with Pneumonia: An Observational Cohort Study
Source: Antibiotics (Basel). 2021 May 11;10(5):557. doi: 10.3390/antibiotics10050557 (PMC8151456; doi:10.3390/antibiotics10050557)
Supplement: Supplementary file 1 [file antibiotics-10-00557-s001.zip › antibiotics-1211732-supplementary/Ceftriaxone_Supplementary materials_File S1.pdf]

Pharmacokinetic/Pharmacodynamic Target Attainment Based on Measured Versus Predicted Unbound Ceftriaxone Concentrations in Critically Ill Patients with Pneumonia: An Observational Cohort Study

Supplementary Materials

File S1: Bioanalytical method

Analytical standards and reagents

Ceftriaxone sodium (pharmaceutical secondary standard, certified reference material, purity = 99.6%), cefazolin, and ammonium formate were obtained from Sigma-Aldrich (Bornem, Belgium). Acetonitrile, purified water, and formic acid were obtained from Biosolve (Valkenswaard, the Netherlands).

Preparation of calibration standards and quality control samples

Stock solutions of ceftriaxone and cefazolin were prepared separately in water and dimethyl sulfoxide at a concentration of 3.0 mg/mL and 1.0 mg/mL, respectively. All stock solutions were kept at -80°C. The working solutions of ceftriaxone were prepared by dilution from the primary stock solution using blank human plasma. The calibration curves, consisting of six calibration standards ranging from 2 µg/mL to 400 µg/mL, were prepared by dilution of the 400 µg/mL ceftriaxone working solution. Low, medium and high-concentration quality control samples (4, 40, 400 µg/mL) were prepared in a similar way. The lower limit of quantification of the bioanalytical method was 2 µg/mL. A 3 µg/mL cefazolin solution (internal standard) was prepared by dilution of the primary stock solution of cefazolin using acetonitrile.

Sample preparation

A total of 50 µL of the plasma study samples were spiked with 50 µL internal standard solution (3 µg/mL). After vortex mixing, mixtures were centrifuged at 8000 rpm for 10 min at 4°C using a Hettich 220R centrifuge (Geldermalsen, the Netherlands). Subsequently, the supernatant was

diluted 10 times with a mixture of mobile phase A and B (9:1; *v*/*v*). The solution was transferred to the autosampler vials.

Liquid chromatography

Liquid chromatography was performed on a Shimadzu Nexera X2 ultra-fast liquid chromatography system (Shimadzu, Kyoto, Japan). The chromatographic separation was achieved using a Cortecs UPLC C18 column (2.1 x 150 mm, 1.6 µm particle size, Waters, Zellik, Belgium), with a gradient elution by a mobile phase consisting of 10 mM ammonium formate (pH 2.5) (A) and acetonitrile with 0.1% formic acid (B). The mobile phase gradient commenced at 10% solvent B which increased slowly to 40% by 1.5 min and was then maintained for 1.00 min. Next, the proportion of solvent B was returned to 10% and maintained until the end of the chromatographic run. The analysis was run at a flow rate of 0.40 ml/min, with the column and sample temperature kept at 40°C and 4°C, respectively. The total run time was 6.5 min and the injection volume was 3 µL. The retention times for ceftriaxone and cefazolin (internal standard) were 3.08 min and 3.25 min, respectively.

Mass spectrometry

The quantitative mass spectrometric analysis was performed on a Shimadzu LCMS-8050 triple-quadrupole mass spectrometer (Shimadzu, Kyoto, Japan). Electrospray experiments were performed in positive ion mode (ES+), with an interface voltage of 4.0 kV. The block and desolvation line (DL) temperatures were 400°C and 220°C, respectively. Purified nitrogen gas was used as the nebulizing gas and the drying gas, with flow rates of 2.00 L/min and 10 L/min, respectively. Argon was used as the collision gas at 270 kPa.

Ceftriaxone and cefazolin were detected using multiple reaction monitoring (MRM) of the following specific transitions: event 1, ceftriaxone (555.05 > 396.10 as quantifier product ion and 555.05 > 324.10 as qualifier product ion); event 2, cefazolin (internal standard) (454.90 > 323.20 as quantifier product ion and 454.90> 156.20 as qualifier product ion). The dwell time for both transitions of ceftriaxone was 72 msec, with collision energies of -14.0 V and -17.0 V, for the quantifier and qualifier product ion, respectively. The dwell time for both MRM transitions of cefazolin was 72 msec with collision energies of -12.0 V and -17.0 V, for the quantifier and qualifier product ion, respectively.

Precision and accuracy

Four different precision and accuracy batches were analysed on four different days. This was performed separately for total ceftriaxone concentrations and ceftriaxone concentrations in plasma and phosphate buffered saline after equilibrium dialysis (ED). The coefficient of variation (CV (%)) and accuracy (%) of QCs at each level within each batch (intra-assay) was within acceptable limits for all batches, except for the HQC in batch 2 of the ED experiment. The amount HQC samples was increased subsequently, and was found to be within acceptable limits for the other batches. Measured ceftriaxone concentrations in batch 2 were not higher than 144 μg/mL (HQC = 320 μg/mL). Mean inter-assay (overall) CV (%) (precision) and accuracy (%) results of all QC replicates are presented in Table 1.

Table 1. Inter-assay (overall) precision and accuracy results

QC ID	$[CEF]_{Total}$		Equilibrium dialysis	
	**Accuracy (%)**	**CV (%)**	**Accuracy (%)**	**CV (%)**
LLOQ QC	94.1	8.4	101.8	9.7
LQC	91.0	10.0	97.9	10.4
MQC	95.0	8.6	91.2	7.2
HQC	99.8	15.2	107.1	19.9

Stability

The autosampler was cooled at 4°C. Based on current literature, no stability issues were expected for ceftriaxone at 4°C. All QCs showed good intraday accuracy and precision, thus confirming appropriate stability.
