# Supplementary material for: Pharmacokinetic/Pharmacodynamic Target Attainment Based on Measured versus Predicted Unbound Ceftriaxone Concentrations in Critically Ill Patients with Pneumonia: An Observational Cohort Study
Source: Antibiotics (Basel). 2021 May 11;10(5):557. doi: 10.3390/antibiotics10050557 (PMC8151456; doi:10.3390/antibiotics10050557)
Supplement: Supplementary file 1 [file antibiotics-10-00557-s001.zip › antibiotics-1211732-supplementary/Ceftriaxone_Supplementary materials_File S2.pdf]

Pharmacokinetic/Pharmacodynamic Target Attainment Based on Measured Versus Predicted Unbound Ceftriaxone Concentrations in Critically Ill Patients with Pneumonia: An Observational Cohort Study

Supplementary Materials

File S2: Linear and nonlinear protein binding equations

Linear protein binding

$$\mathrm{CEFt} = \mathrm{CEFu} + \mathrm{CEFt}\ \times \mathrm{b} \qquad \text{(Eq. S1)}$$

where CEFt stands for total ceftriaxone concentration (mmol/L), CEFu stands for unbound ceftriaxone concentration (mmol/L), b stands for the linear protein binding constant.

Nonlinear protein binding

$$EFt = CEFu + CEFu \times \frac{(Bmax\ \times \left(\frac{ALB}{median\ ALB}\right)^{h})}{(B50 + CEFu)} \qquad \text{(Eq. S2)}$$

where CEFt stands for total ceftriaxone concentration (mmol/L), CEFu stands for unbound ceftriaxone concentration (mmol/L), Bmax stands for maximum binding capacity (mmol/L), ALB stands for albumin concentration (mmol/L), median ALB stands for the median albumin concentration (mmol/L) in the study population, h stands for Hill coefficient, and B50 stands for dissociation constant (mmol/L).
