# Supplementary material for: Pharmacokinetic/Pharmacodynamic Target Attainment Based on Measured versus Predicted Unbound Ceftriaxone Concentrations in Critically Ill Patients with Pneumonia: An Observational Cohort Study
Source: Antibiotics (Basel). 2021 May 11;10(5):557. doi: 10.3390/antibiotics10050557 (PMC8151456; doi:10.3390/antibiotics10050557)
Supplement: Supplementary file 1 [file antibiotics-10-00557-s001.zip › antibiotics-1211732-supplementary/Ceftriaxone_Supplementary materials_Table S1.pdf]

Pharmacokinetic/Pharmacodynamic Target Attainment Based on Measured Versus Predicted Unbound Ceftriaxone Concentrations in Critically Ill Patients with Pneumonia: An Observational Cohort Study

Supplementary Materials

Table S1

Table S1. Parameters of the protein binding models used to predict unbound ceftriaxone concentrations.

	Fixed protein binding	Capacity Constant (nP)	Binding affinity constant (k_{aff})	Maximum binding capacity (Bmax)	Hill coefficient (h)	Dissociation constant (B50)
**Fixed average protein binding**	89.5 %	-	-	-	-	-
**Estimated saturable concentration-dependent protein binding, Eq. 4**	-	517 µmol/L	0.0367 L/µmol	-	-	-
**Predicted protein binding, present study, Eq. 2**	-	-	-	−0.64 mmol/L	0.26	1.09 mmol/L
**Predicted protein binding, Bos**	-	-	-	0.12 mmol/L	1.3	0.0092 mmol/L
**Predicted protein binding, Leegwater**	-	-	-	113 mg/L	0.0399	11.5 mg/L
